# The Effects of Tai Chi on Markers of Atherosclerosis, Lower-limb Physical Function, and Cognitive Ability in Adults Aged Over 60: A Randomized Controlled Trial

**DOI:** 10.3390/ijerph16050753

**Published:** 2019-03-01

**Authors:** Shengwen Zhou, Yanjie Zhang, Zhaowei Kong, Paul D. Loprinzi, Yang Hu, Jiajie Ye, Shijie Liu, Jane Jie Yu, Liye Zou

**Affiliations:** 1 Department of Chinese Martial Arts, College of Sport Science, Hunan University of Science and Technology, Yongzhou 425100, China; shengwen1224@huse.edu.cn; 2 Health and Exercise Science Laboratory, Institute of Sports Science, Seoul National University, Seuoul 08826, Korea; elite_zhangyj@163.com; 3 Faculty of Education, University of Macau, Macao, China; zwkong@um.edu.mo; 4 Department of Health, Exercise Science and Recreation Management School of Applied Sciences, The University of Mississippi, Oxford, MS 36877, USA; pdloprin@olemiss.edu; 5 Sports Science Research Center, Beijing Sport University, Beijing 100084, China; huyang@bsu.edu.cn; 6 Department of Rehabilitation Sciences, The Hong Kong Polytechnic University, Hunghom, Hong Kong, China; ye.j.ye@connect.polyu.hk; 7 Department of Physical Education, Wuhan University of Technology, Wuhan 430070, China; liushijie0411@whut.edu.cn; 8 Sports and Exercise Psychology Laboratory, Department of Sports, Science and Physical Education, The Chinese University of Hong Kong, Shatin, New Territories, Hong Kong, China; 9 Lifestyle (Mind-Body Movement) Research Center, College of Sports Science, Shenzhen University, Shenzhen 518060, China

**Keywords:** Tai Chi, mind-body exercise, cognition, balance, arterial stiffness, aging

## Abstract

*Objective*: The purpose of this study was to investigate the effects of Tai Chi (TC) on arterial stiffness, physical function of lower-limb, and cognitive ability in adults aged over 60. *Methods*: This study was a prospective and randomized 12-week intervention trial with three repeated measurements (baseline, 6, and 12 weeks). Sixty healthy adults who met the inclusion criteria were randomly allocated into three training conditions (TC-24, TC-42, and TC-56) matched by gender, with 20 participants (10 males, 10 females) in each of the three groups. We measured the following health outcomes, including markers of atherosclerosis, physical function (leg power, and static and dynamic balance) of lower-limb, and cognitive ability. *Results:* When all three TC groups (p < 0.05) have showed significant improvements on these outcomes but overall cognitive ability at 6 or 12 weeks training period, TC-56 appears to have superior effects on arterial stiffness and static/dynamic balance in the present study. *Conclusions*: Study results of the present study add to growing body of evidence regarding therapeutic TC for health promotion and disease prevention in aging population. Future studies should further determine whether TC-42 and TC-56 are beneficial for other non-Chinese populations, with rigorous research design and follow-up assessment.

## 1. Introduction

As of 2019, the percentage of aging population is increasing rapidly due to improvements in health care, medical technology, and standard of living around the world [1]. Health experts have predicted that the proportion of global population aged over 60 will double from 11% to 22% between 2000 and 2050 [2], and that inevitable aging will challenge the global health care systems over the coming decades [3]. Particularly for older adults, health and longevity are always expected, but at present they tend to be more vulnerable to chronic diseases across their later lifespan [4]. Fall-related injuries and deaths are also prevalent in this age group [4]. Atherosclerosis, as a type of atherosclerotic cardiovascular disease, can be easily detected among people over 60 years old, eventually leading to 50% of deaths in the western world [5]. For example, we recently demonstrated that, among older adults, cardiovascular disease risk profile and low cognitive function were individually and additively related to mortality risk [6]. Relatedly, a normal process of aging has been well documented to associate with cognitive decline or impairment, which has been identified as a risk factor for reduced physical function and activity of daily living [7,8,9]. Briefly speaking, the above mentioned situations are inherently related to the advanced age. Despite the age-associated issues, studies suggest that older adults in general can make great efforts to attenuate the normal aging process through anti-aging herbal medicine [10], medical devices [11,12] and/or exercise [13,14]. 

Tai Chi (TC) as a type of moderate-intensity exercise is rooted in traditional Chinese philosophy of Taoism and medicine, and it can offer good health-promoting effects for aging adults [15,16,17]. TC was first known by ancient Chinese people, and it dates back to at least three thousand years ago [18,19,20]. With the passage of time, five mainstream types of TC (Chen style, Yang style, Sun style, Hao style, and Wu style) have been further developed [21,22]. Nevertheless, these different TC styles share common elements; breathing control, skeletal muscle extension/flexion, body awareness, mental concentration, and whole-body coordination [23,24]. In terms of TC forms, it presents a variety of forms such as 8-form, 16-form, 24-form, 42-form, 56-form and even 108-form [24,25,26,27,28]. Each TC style or form has its own unique features. To promote TC globally, Chinese TC experts have carefully selected signature movements from 88-form TC and developed 24-form Yang-style. Similarly, 42-form TC (TC-42) is comprised of integrated classic movements of Yang, Chen, Wu, and Sun styles, characterized by smooth and coherent movements. The 56-form Chen style TC (TC-56) is one of the oldest styles in China, and contains unique properties such as silk reeling training, hardness and softness, loose and vibration and jumping at multiple directions. TC-56 also involves more strength, endurance and flexibility exercises, which makes it easy to attract a large number of practitioners. 

TC is a safe exercise program that has been considered to be a novel strategy contributing numerous health benefits for aging adults [29,30]. With regard to physical function, for example, a randomized controlled trial by Taylor-Piliae et al. [31] investigated the effects of TC-24 in older adults, suggesting that 12-week (60 min per session, three times per week) TC training enhanced balance and mobility [23]. Jain and colleagues [32] found that increased muscle strength and mobility following a 10-week TC training was associated with enhanced overall health. TC training has been shown to be an effective therapy to maintain or elevate arterial compliance for older adults [33]. Shin et al. [34] found that a 10-week (3 sessions x 60 min weekly) program of TC increased arterial compliance among female seniors. Mechanistically, these results suggest that TC can prevent cardiovascular diseases through improving endothelial function and arterial stiffness [34]. Beyond physical function and chronic illness, it is notable that early works have shown that TC has a positive effect on preventing cognitive decline. For example, Taylor-Piliae et al. [35] found that older community dwellers who practiced TC-24 for 12 months improved their cognitive function (i.e., executive function, work memory). This observation was supported by Siu et al. [36], indicating that TC-24 is considered to be an effective therapy to improve cognition. In sum, prior research demonstrates that TC may be safe effective in improving physical and mental health of the elderly. 

However, despite the benefits of TC training being widely accepted, as the diversity of TC exercise and the studies comparing the effects of different TC styles are lacking, it is difficult to justify which one style of TC has more benefits than other forms. The objective of this current study, therefore, was designed to determine whether 12 weeks of three styles of TC intervention (three times weekly, with each session lasting 60 min) have similar effects on physical function, atherosclerosis, and cognitive function among older adults. These findings will provide important information as to which style of TC intervention is more effective for older adults.

## 2. Methods 

**Figure 1:**
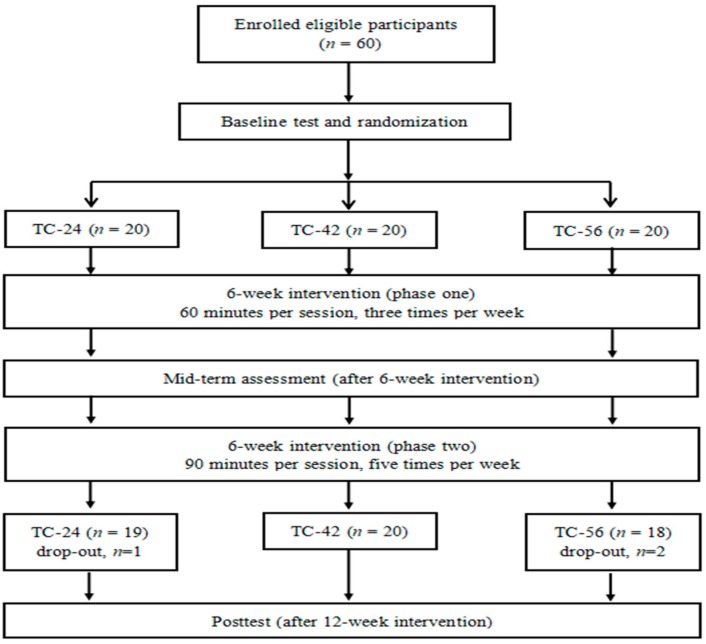
Experimental procedures from recruitment to the end of experimental design.

This study was a prospective, randomized 12-week intervention trial with three repeated measurements (baseline, 6, and 12 weeks). Three styles of TC, including TC-24, TC-42, and TC-56 were conducted as intervention conditions. The study was approved by the ethics committees of university. Study procedures are presented in Figure 1. 

### 2.1. Study Participants

A total of 60 Chinese adults (age range: 60-79 years old; mean age: 65.6 ± 4.6 years) were recruited and participated in this study voluntarily. Participants were considered eligible if they met the following inclusion criteria: (1) male or female aged 60 years or above; (2) healthy and able to participate in exercise; (3) normal cognitive function, as indicated by the Mini-Mental State Examination score of ≥ 26); and (4) had not regularly practiced TC or other exercise previously. Subjects who had any serious diseases (e.g., cardiovascular disease, sequela of apoplexy), a history of drug, or alcohol abuse were excluded. All participants were randomly allocated into three training conditions (TC-24, TC-42, and TC-56) matched by gender, with 20 participants (10 males, and 10 females) in each of the three groups. Three subjects (one in the TC-24 group and two in the TC-56 group) dropped out from the intervention. Therefore, the remaining 57 subjects were included for data analyses. All subjects were asked to maintain their usual routine such as diet and activities throughout the study. 

### 2.2. Three Different Styles of Tai Chi Intervention

The intervention duration was 12 weeks. Subjects in the three groups (TC-24, TC-42, and TC-56) were instructed to perform one specific style of TC program accordingly. In phase one (Week 1-6), they attended three 60-min exercise sessions per week for six weeks. Each session consisted of 10 min of warm-up, 40 min of TC practice including learning of new content, and 10 min of cool-down. In phase two (Week 7-12), the frequency and duration of practice were increased. During these last 6 weeks, subjects performed five 90-min exercise sessions per week. In each session, there were 70 min of TC practice except for a 10-min warm-up and 10-min cool-down. The intervention was led by an experienced TC instructor. The movements required in different intervention conditions were explained and demonstrated by the same TC instructor who had more than 15 years of teaching experience, and subjects followed and performed practice independently afterwards.

### 2.3. Outcome Measures

#### 2.3.1. Demographic and Anthropometric Information

Demographic information including date of birth and sex were reported by the subjects. Body height and weight (Healthometer 402KL Beam Scale weight/ Height Rod, PELSTAR, McCook, IL, USA), and resting heart rate of each subject were assessed prior to the intervention.

#### 2.3.2. Atherosclerosis

Resting heart rate, Resting ankle brachial index (ABI) and ankle pulse wave velocity (PWV) were measured by an oscillometric method using a VaSera VS-1500A (Fukuda Denshi, Tokyo, Japan) as markers of atherosclerosis. This method has shown to be valid in the elderly [37]. Similar to our previously published work [38], after the subjects had rested in the supine position for 5 min, pneumatic pressure cuffs were placed around their both arms and both ankles before measurements. The device produced an electrocardiogram and simultaneously recorded the ABI, PWV, the brachial and ankle blood pressures on the left and right sides, and the heart sounds. ABI suggests the blockage of the arteries in legs with normal ABI ranging from 1.0 to 1.4. PWV measures arterial stiffness which is an independent predictor of cardiovascular risk with a lower PWV score representing better vascular elasticity [39].

#### 2.3.3. Cognitive Ability

Cognitive ability of the subjects was assessed using the Chinese version of the Montreal Cognitive Assessment (MoCA) [40]. The MoCA is a 30-item test including various components of cognition such as executive function, language, orientation, memory, and abstraction.MoCA is scored from 0 to 30, with a higher score representing a better cognitive ability. This measure has good criterion-related validity (Pearson correlation coefficient MoCA vs MMSE = 0.787) and reliable internal consistency (Cronbach alpha = 0.807) and has been widely used to assess mild cognitive impairment in older adults [41]. 

#### 2.3.4. Physical Function in Lower Limbs

##### Chair Rise Performance Test 

A standard chair without arms (approximate seat height of 45 cm) was placed against a wall. The subjects were seated with their back in an upright position and their feet flat on the floor. They were asked to stand completely up and then completely back down as fast as possible after the “1, 2, 3, go” command with their arms folded across the chest. The outcome measure was the time it took them to continuously perform five complete chair stands. All subjects had three attempts with an approximate 3 min break in between. The best (fastest) score was selected for analysis [42]. All subjects were performed this test using the same chair and with similar ambient conditions. 

##### Balance

Static balance was assessed by one-leg standing test. With their eyes closed, the subjects were asked to stand on their preferred leg, lift the knee of the other leg to approximately 90°, keeping their arms by their sides, and maintain balance without using any assistive device. The test was over when the stance foot shifted or when the lifted foot was replaced on the ground, whichever occurred first. Both legs were tested successively. The duration of standing (in second) was recorded, with a longer duration representing better static balance [43].

##### The 6-Meter Walk Test (6MWT)

A smooth and flat 12-metre walkway was marked out on the floor with tape markers being placed at the 0-, 3-, 9-, and 12-m points along the walkway. Standing at the 0-metre point, subjects were asked to walk to the end of the walkway (marked at 12-m point) at their own comfortable speed without stopping. A tester timed the subjects over the 6 m using a stopwatch. The outcome measure of this test was the time spent walking in seconds (s) [44]. The first and last 3 m of the walk were not timed, due to changes in velocity that occurs when people start and stop walking. All subjects were limited to two attempts with the best attempt being used in the analyses.

##### Timed Up and Go Test (TUG)

The TUG test measures the time, in seconds (s), taken by the subject to stand up from a standard arm chair (approximate seat height of 45 cm), walk three meters, turn around, walk back to the chair and sit down. The test was performed without physical assistance. The subject was instructed to be seated on the chair with their arms resting on the chair’s arms, and to stand up on the word “go” command. The stopwatch was started on the “go” command and stopped as the subject sat down [45]. Each subject had three attempts with the best attempt being recorded.

### 2.4. Data Analysis

The software package SPSS 22.0 (SPSS Inc. Chicago, IL, USA) was used for data analyses. Descriptive statistics such as mean and standard deviation were presented where appropriate. Chi-square statistic was used to compare the number of subjects in the three groups by sex. One-way factorial univariate analysis of variance (ANOVA) was used to compare all baseline data among the three groups. One-way (Group: TC-24, TC-42, TC-56) factorial univariate analysis of covariance (ANCOVA) with repeated measures (Time: baseline, 6-week, 12-week) was used to compare the intervention effects among groups after controlling for age. In this analysis, to increase statistical power and precision, baseline score was adjusted as a covariate when a significant group difference existed at baseline. Analyses of simple effects and post hoc Bonferroni adjustments were performed when significant main and interaction effects were confirmed. Statistical significance was set at p < 0.05 for all tests.

## 3. Results

### 3.1. Baseline Data

For heart rate, there was a significant group difference with the TC-42 group having significantly lower heart rate when compared to the TC-24 (p < 0.05) and TC-56 groups (p < 0.01). When compared to the TC-42 and TC-56 groups, the TC-24 group had higher scores in PWV on both ankles and left leg standing, indicating that the TC-24 group had poorer vascular elasticity and better balance than the other two groups (all p < 0.05). Additionally, the TC-42 group had a higher score in chair stand test (p < 0.05) and a shorter time in 6-MWT (p < 0.05) than the TC-24 group, suggesting that the TC-42 group had better functional ability than the TC-24 group at baseline. No significant differences were evident in age, BMI, MoCA, ABI, one-leg standing (right leg), and TUG among groups at baseline (all p > 0.05). Age was adjusted as a covariate in all the subsequent analyses with considering its impacts on all outcomes in this study. Heart rate, PWV, left leg standing, chair stand test score, and 6-MWT score were thus treated as covariates in the subsequent analyses where appropriate. No significant gender differences were found in any outcome measures. Therefore, the data were not analyzed separately by sex. All outcome measures across groups at baseline are presented in Table 1. 

### 3.2. Intervention Effects

#### 3.2.1. Atherosclerosis

Results of ANCOVAs showed that a significant *Group* by *Time* interaction effect in ABI for left leg, F (4, 104) = 2.632, p < 0.05, in which ABI significantly decreased in the TC-42 (p < 0.001) and TC-56 (p < 0.05) groups at Week 6 when compared to baseline. There was a significant main effect of *Group*, F (2, 51) = 3.951, p < 0.05 on PWV for left ankle; however, no significant group differences were evident in post-hoc tests. No significant main and interaction effects were found in ABI for right leg and PWV for right ankle.

#### 3.2.2. Cognitive Ability

No significant main and interaction effects were observed for MoCA.

#### 3.2.3. Chair Rise Performance Test

No significant main and interaction effects were observed for chair rise performance.

#### 3.2.4. Balance

In the one leg standing test, there was a significant *Group* main effect, F (2, 51) = 15.628, p < 0.001 on left-leg, with the TC-56 group standing for a longer time than the TC-24 and TC-42 groups (both p < 0.001). A significant *Group* by *Time* interaction effect was found for left-leg, F (2, 51) = 4.888, p < 0.05. Specifically, in both TC-42 and TC-56 groups, the subjects performed a longer duration with their left-leg at Week 12 than that at Week 6 (both p < 0.001). No significant main and interaction effects were found for right leg. All results are presented in Table 2.

#### 3.2.5. MWT Test

Significant main effects on *Group*, F (2, 51) = 8.188, p < 0.05, and *Time*, F (1, 51) = 7.122, p < 0.05 were found, in which the TC-42 and TC-56 groups performed the task faster when compared to the TC-24 group (both p < 0.05) post intervention after controlling for the baseline score; and time spent in the 6-MWT was shorter at Week 6 than that at Week 12 in general (p < 0.05).

#### 3.2.6. TUG Test

There was a significant main *Time* effect, F (2, 104) = 5.581, p < 0.01, in which the score significantly and progressively decreased from baseline to Week 6 and further to Week 12 (all p < 0.001) regardless of group allocation. All results are presented in Table 2.

## 4. Discussion

The aim of this study was to investigate the effects of three different styles of TC on indices of atherosclerosis, lower-limb physical function, and cognitive ability in healthy adults aged over 60. Results of the present study indicate that all three (24-form, 42-form, and 56-form) TC styles are effective in improving various health-related outcomes. Furthermore, both TC-56 and TC-42 seem to be superior to TC-24 in ABI test, Stork Balance test, and 6-MWT, at least in the present study. None of the participants of the three TC groups reported adverse events, indicating that both TC-42 and TC-56 are feasible exercise intervention programs to help the elderly maintain well-being, while minimizing safety concerns. More detailed information will be discussed in that narrative that follows.

### 4.1. Ankle Brachial Index and Ankle Pulse Wave Velocity

ABI and PWV are indices of atherosclerosis and arterial stiffness [46]. It is well accepted that people with arterial stiffness have greater development of cardiovascular disease through the process of atherosclerosis [47]. Particularly, increased arterial stiffness is commonly reported in aging populations, which increases the risk for hypertension [48]. Globally, the increasing number of atherosclerotic cardiovascular disease (ACD) has contributed to social economic burden, which has become a major public health challenge [49]. Thus, increased efforts are needed to reduce arterial stiffness in order to effectively prevent ACD. Regular aerobic exercises as part of a healthy lifestyle has been shown to be an effective intervention for preventing and treating arterial stiffness [50].

In the present study, we found that both TC-42 and TC-56 showed significantly greater reduction of ABI of left leg following the 6-week training, but this positive result was not observed in TC-24. In addition, all groups showed significant lower values in PWV on both ankles at Week 12 than that at Week 6, indicating that all subjects had significant improvements in vascular elasticity after receiving training in phase two. Taken together, all three TC styles have the potential to effectively improve indices of atherosclerosis and arterial stiffness, which is supported by a previous study investigating the beneficial effects of 3-months of TC training on arterial stiffness in older women with rheumatoid arthritis [34]. One possible explanation is that TC can effectively modulate heart rate variability and baroreflex sensitivity, which are highly related to arterial stiffness observed in aging population [23,51,52]. Of note, TC-42 and TC-56 are able to trigger initial intervention effects on ABI at week 6 (minimal effective dose) while this positive effect was not observed in TC-24. This may be attributed to the greater complexity of movements in TC-42 and TC-56, as compared to TC-24 including fundamental movements.

### 4.2. Cognitive Ability

All three TC groups demonstrated progressively increased scores from Week 6 to Week 12 even if their significant levels were not reached. This suggests that the TC type does not moderate the beneficial effects of TC on cognitive function. These results are consistent with conclusions reached in recent review papers [53,54,55]. Overall improvement in cognitive ability may be attributed to the following reasons. First, mastery of continuous movement sequences in TC potentially alters synaptic plasticity, which may improve short-term memory function. Second, TC typically involves dynamic weight-shifting at multiple directions. To effectively stabilize the whole body during TC performance, some advanced cognition are needed, such as planning, information processing speed, attentive ability, and visual-spatial ability [56]. These movement features provide additional cognitive stimulation. In the present study, instructor-led training mode was applied, which can potentially facilitate communication among trainees, peers and instructor. This social interaction requires intellectual engagement so that it can further benefit cognitive ability through emotion regulation and peer group support [57]. Therefore, TC could be considered an early intervention to prevent cognitive decline in aging populations. However, it is worth noting that non-signficanct results on overall cognitive ability may be primarily attributed to that MoCA as a diagnostic tool is not sentitive to TC exercise intervention. Future studies should focus on specific cognition-related outcome measures such as the Trail Making Test A (information processing speed, attentional ability) and B (task switing and executive function), the Rey Complex Figure (short term memory), and 2-N back test (working memory).

### 4.3. Physical Function in Lower Extremity

In the SBT, we observed that on left-leg, TC-56 group showed significantly longer time than the TC-24 and TC-42 groups. Furthermore, participants of both TC-42 and TC-56 groups showed significantly better performance with their left-leg at Week 12 than that at Week 6; significantly and progressively prolonged their standing time with their right-leg from baseline to Week 6 and then to Week 12. However, only a significant improvement on right-leg standing was observed from baseline to Week 6 in the TC-24 group. Taken together, while three TC styles are effective at improving static balance of adults aged over 60, we observed that TC-56 can achieve the maximum intervention effect on static balance as compared to the two other TC styles (TC-42 is more effective than TC-24). Furthermore, this positive effect was increased as intervention duration was prolonged. Similar findings were observed for 6-MWT. Of note, we observed that for both TC-56 and TC-42, time spent in the 6-MWT was shorter at Week 6 than that at Week 12 in general, suggesting that 6-weeks may be a sufficient intervention dose to elicit positive responses from TC-56 and TC-42.

For the TUG test, performance was significantly and progressively improved from baseline to Week 6 and further to Week 12 in all three TC groups. Results in a recently published review paper [25] indicated that TC is an effective intervention to maintain and improve lower limb proprioception in adults older than 55. This improved prioprioception may partially explain the findings of the present study. In addition, accumulating evidence indicates that cognitive function is closely associated with balance performance [58,59,60]. Improved overall cognition in the present study may play a substantial role in enhancing balance performance. However, in the present study, we did not observe significant improvements on leg power (as measured by the CRPT) even in TC-56, which involves skipping movements.

### 4.4. Strength and Limitations

The present study included several strengths: (1) predetermined eligibility criteria; (2) similar demographic information (gender, age, race, and maturation stage) of participants in each TC group; (3) randomized controlled design; (4) the same TC expert to deliver the intervention protocol; (5) use of standard assessment tools; (6) use of ANCOVA to control for confounding factors; and (7) assessors were blinded to group allocation. A number of limitations in the present study need to be acknowledged as well. First, both participants and TC instructor were not blinded to the purpose of the present study, which typically exists in nonpharmacological interventional studies. Participants in the TC-56 possibly have greater expectations for improvement. Second, even though the same TC instructor can eliminate personality biases, it may also increase the possibility of information contamination across groups. Third, participants were all Chinese in the present study, limiting generalizability to other non-Chinese populations. Fourth, given the difficulty of participant recruitment, we did not use a non-active control group. Fifth, follow-up assessments were not implemented in the present study, so we are unsure how long TC intervention effects last, especially TC-42 and TC-56. Sixth, metabolic expenditure was not measured during performance, thus we cannot determine whether the superior effect of TC-56 is attributed to energy expenditure.

## 5. Conclusions

This is the first study to compare the effects of three different styles of TC on indices of atherosclerosis, physical function of lower-limb, and cognition among adults aged between 60 and 79 years. Results of the present study add to the growing body of evidence regarding the therapeutic benefits of TC for health promotion and disease prevention in aging populations. While all three TC styles showed beneficial effects on various health outcomes, TC-56 had superior effects on arterial stiffness and static/dynamic balance. Future studies should further determine whether TC-42 and TC-56 are beneficial for other non-Chinese populations, with rigorous research design and follow-up assessments.

## Figures and Tables

**Table 1:** All outcome measures across groups at baseline (mean±SD).

	TC-24 Group (*n* = 19)	TC-42 Group (*n* = 20)	TC-56 Group (*n*= 18)	Statistics
**Demographic and anthropometric characteristics**				
Age (year)	67.4±5.9	64.9±3.5	64.5±3.6	2.393
Sex (male/female, *n*)	9/10	10/10	9/9	0.035
BMI (kg/m^2^)	23.4±2.9	24.6±2.3	25.0±2.6	2.250
Resting heart rate (beats/minute)	76.1±8.6	71.0±4.9	77.3±4.2	5.638 **
**Outcome variables**				
ABI				
*left side*	1.2±0.1	1.2±0.1	1.2±0.1	0.515
*right side*	1.2±0.1	1.1±0.1	1.2±0.1	0.962
PWV				
*left ankle*	7.2±1.3	6.1±0.7	6.2±0.8	6.762 **
*right ankle*	7.2±1.3	6.1±0.7	6.4±0.6	7.227 **
MoCA	26.5±1.5	26.0±1.4	25.8±1.5	1.107
Chair rise performance test	9.9±2.4	8.3±1.2	9.8±2.2	4.049 *
One leg standing test				
*left leg*	4.7±3.0	1.9±1.1	3.0±1.7	9.070 ***
*right leg*	3.3±2.7	2.3±1.8	3.7±2.2	2.028
6MWT	5.6±1.4	4.8±0.5	5.0±0.5	4.666 *
TUG	11.7±2.9	10.5±1.6	11.2±2.8	1.243

Note: BMI = body mass index; ABI = ankle brachial index; PWV = pulse wave velocity; MoCA = the Chinese version of the Montreal Cognitive Assessment; 6MWT = The 6-m walk test; TUG = Timed up and go test. * *p* < 0.05; ** *p* < 0.01, *** *p* < 0.001.

**Table 2:** Descriptive statistics of all outcome variables across groups at mid-term (Week 6) and post (Week 12).

	TC-24 Group (*n* = 19)		TC-42 Group (*n* = 20)		TC-56 Group (*n* = 18)		*F* Value ^#^		
	6-week	12-week	6-week	12-week	6-week	12-week	Group	Time	Group X Time
ABI									
*left side*	1.2±0.1	1.2±0.1	1.1±0.1	1.2±0.1	1.2±0.1	1.1±0.1	0.239	1.134	2.632 *
*right side*	1.1±0.1	1.1±0.1	1.1±0.1	1.1±0.2	1.1±0.1	1.1±0.1	0.218	0.065	0.453
PWV									
*left ankle*	7.1±1.2	6.8±1.0	6.1±0.7	5.9±0.6	6.1±0.7	5.9±0.6	3.951 *	3.182	1.465
*right ankle*	7.1±1.2	6.9±1.2	6.0±0.7	5.9±0.7	6.3±0.6	6.0±0.5	1.699	1.423	1.796
MoCA	28.2±1.3	29.8±0.5	27.9±1.6	29.8±0.6	27.7±1.6	29.6±0.7	1.697	2.337	1.479
Chair rise performance test	9.5±2.3	8.8±1.7	7.9±1.2	7.6±1.0	9.2±2.0	8.9±1.8	0.168	0.480	2.013
One leg standing test									
*left leg*	5.5±3.0	5.7±3.3	2.4±1.2	3.2±1.0	4.2±2.3	5.3±1.8	15.628 ***	1.160	4.888 *
*right leg*	4.3±2.6	4.5±2.3	2.8±2.3	3.8±2.4	5.2±2.7	6.2±2.2	3.099	1.491	2.289
6MWT	5.4±1.3	5.7±1.1	4.4±0.5	4.5±0.5	4.5±0.6	4.7±0.4	8.188 **	7.122 *	3.137
TUG	11.3±2.8	10.4±2.1	10.0±1.5	9.0±1.3	10.7±2.7	9.3±1.9	1.024	5.581 **	1.052

^#^ After controlling for age, resting heart rate, and the score of PWV, left-leg standing test, chair rise performance test, and 6-MWT whenever appropriate. * *p* < 0.05; ** *p* < 0.01, *** *p* < 0.001.
